# Crystal structure of a polymeric calcium levulinate dihydrate: *catena*-poly[[di­aqua­calcium]-bis­(μ_2_-4-oxo­butano­ato)]

**DOI:** 10.1107/S2056989015006696

**Published:** 2015-04-18

**Authors:** Ananda S. Amarasekara, Dominique T. Sterling-Wells, Carlos Ordonez, Marie-Josiane Ohoueu, Marina S. Fonari

**Affiliations:** a Department of Chemistry, Prairie View A&M University, Prairie View, TX 77446, USA; b Department of Natural Sciences, New Mexico Highlands University, Las Vegas, NM 87701, USA; c Institute of Applied Physics, Academy of Sciences of Moldova, Academy Str. 5, MD2028, Chisinau, Republic of Moldova

**Keywords:** crystal structure, coordination polymer, calcium levulinate dihydrate, levulinic acid, hydrogen bonding

## Abstract

In the title calcium levulinate complex, [Ca(C_5_H_7_O_3_)_2_(H_2_O)_2_]_*n*_, the Ca^2+^ ion lies on a twofold rotation axis and is octa­coordinated by two aqua ligands and six O atoms from four symmetry-related carboxyl­ate ligands, giving a distorted square-anti­prismatic coordination stereochemistry [Ca—O bond-length range = 2.355 (1)–2.599 (1) Å]. The levulinate ligands act both in a bidentate carboxyl *O*,*O*′-chelate mode and in a bridging mode through one carboxyl O atom with an inversion-related Ca^2+^ atom, giving a Ca⋯Ca separation of 4.0326 (7) Å. A coordination polymeric chain structure is generated, extending along the *c*-axial direction. The coordinating water mol­ecules act as double donors and participate in intra-chain O—H⋯O hydrogen bonds with carboxyl O atoms, and in inter-chain O—H⋯O hydrogen bonds with carbonyl O atoms, thus forming an overall three-dimensional structure.

## Abstract 2

The crystal structure of calcium levulinate dihydrate forms a one-dimensional coordination polymer based on a CaO_8_ complex unit which lies on a twofold rotation axis. This unit comprises two monodentate water O-atom donors and six carboxyl­ate O-atom donors, two of which are also bridging, from the two bidentate chelate levulinate ligands. The complex chains are stabilized by intra- and inter­molecular water O—H⋯O hydrogen bonds, forming an overall three-dimensional structure.

## Chemical context   

Levulinic acid (4-oxo­penta­noic acid) is a biomass-derived keto acid and is a potential precursor for renewable fuels as well as polymeric materials (Mukherjee *et al.*, 2015 ▸). A number of metal salts of levulinic acid have been prepared for a variety of applications and the calcium salt with formula Ca(C_5_H_7_O_3_)_2_·2H_2_O is the most widely studied levulinate, as it has been used for over 80 years as a calcium supplement (Proskouriakoff, 1933 ▸). The revived inter­est in calcium levulinate is due to a recent discovery that pyrolysis of this readily accessible renewable biomass-based calcium salt can be used to produce biofuels *via* a ketonic deca­rboxylation process with recycling of calcium as CaCO_3_ (Schwartz *et al.*, 2010 ▸; Case *et al.*, 2012 ▸). In addition, we have recently shown that acid-catalyzed hydro­thermal degradation of cellulose and neutral­ization of the filtrate with calcium hydroxide can be used to prepare a mixture of calcium levulinate and calcium formate and the pyrolysis of this mixture at 623 K can be used to produce γ-valerolactone (Amarasekara *et al.*, 2015 ▸). Recently, Bryce and co-workers published the solid-state ^13^C NMR spectrum of calcium levulinate in which they identified only one type of a levulinate anion (Widdifield *et al.*, 2014 ▸). However, there are no reports on X-ray crystallographic studies on this well known calcium carboxyl­ate. Our inter­est in thermal properties and biofuel applications of calcium levulinate has led us to study the structure of this salt and in this communication we report the crystal structure of calcium levulinate dihydrate, [Ca(C_5_H_7_O_3_)_2_(H_2_O)_2_]_*n*_.
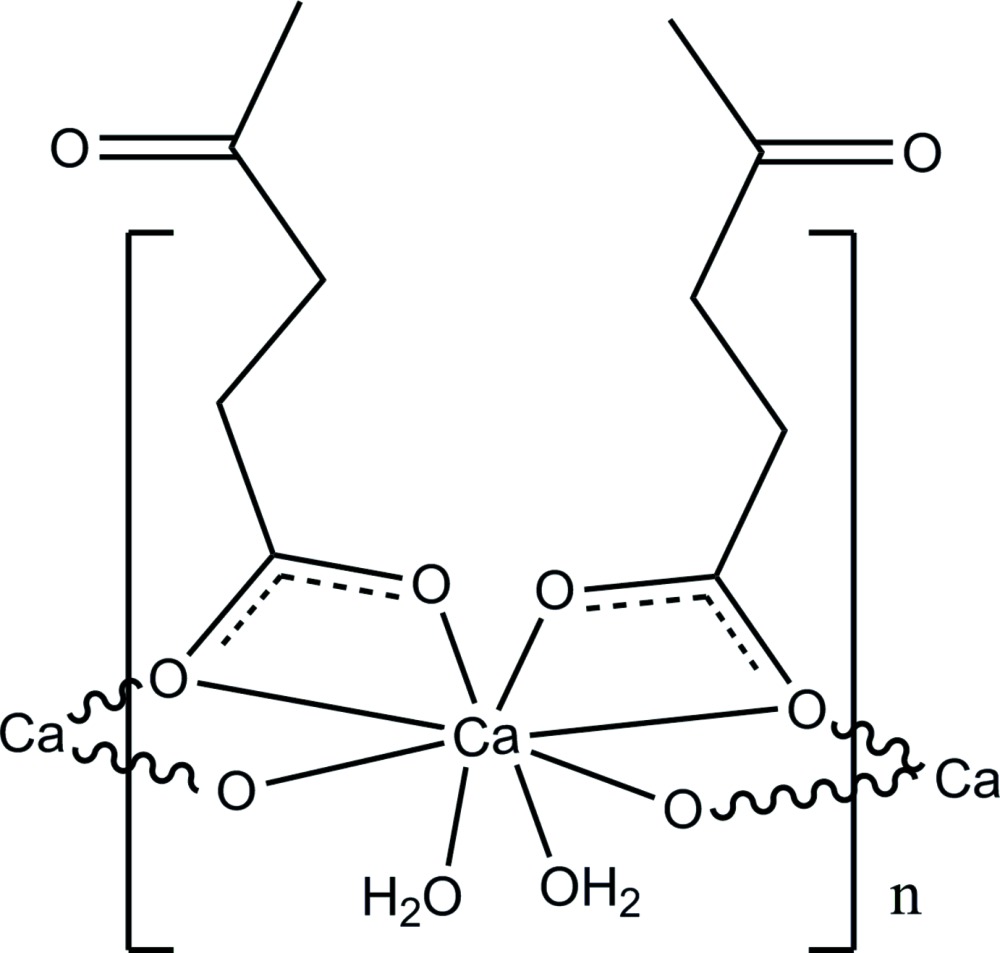


## Structural commentary   

**Figure 1:**
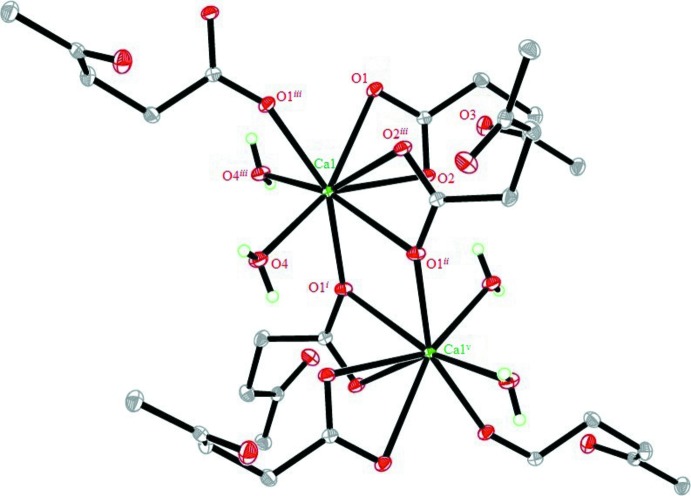
A portion of the crystal structure of the title complex, displaying the atomic labeling. Displacement ellipsoids are drawn at the 50% probability level. Symmetry code (v): −*x* + 1, −*y*, −*z*; for other codes, see Table 1 ▸.

**Figure 2:**
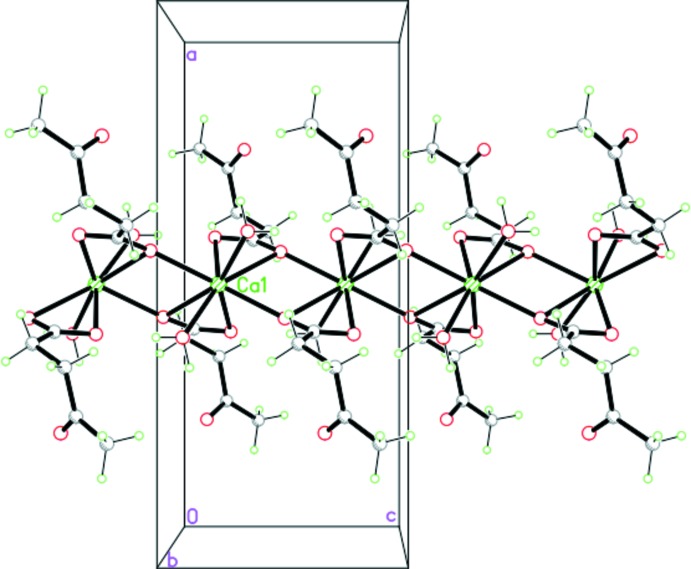
The one-dimensional coordination polymeric chain extending along the *c* axis.

The calcium levulinate structure contains one Ca^2+^ cation, two levulinate anions and two water mol­ecules per formula unit, with the Ca^2+^ cation situated on a twofold rotation axis (Fig. 1 ▸). The cation is octa­coordinated and exhibits a distorted square anti­prismatic stereochemistry with Ca—O bond lengths in the range of 2.355 (1)–2.599 (1) Å (Table 1 ▸). The levulinate carboxyl O atoms (O1 and O2) coordinate to Ca^2+^ cations in two coordination modes, a bidentate *O*,*O*′-chelate mode and a bridging mode through O1^i^ with an inversion-related Ca^2+^ centre, giving a Ca1⋯Ca1^i^ or Ca1⋯Ca1^v^ separation of 4.0326 (7) Å [for symmetry code (i) see Table 1 ▸; symmetry code (v): −*x* + 1, −*y*, −*z*]. Furthermore, due to this type of coordination environment, the two levulinate anions are almost perpendicular to each other, with an O2—Ca1—O2^iii^ angle = 75.78 (5)° [for code (iii), see Table 1 ▸]. The extended one-dimensional coordination polymeric chain generated lies parallel to the *c* axis (Fig. 2 ▸) and within each chain, the coordinating water mol­ecules form intra-chain O4—H4*B*⋯O2^v^_carbox­yl_ hydrogen-bonds (Table 2 ▸).

## Supra­molecular features   

**Figure 3:**
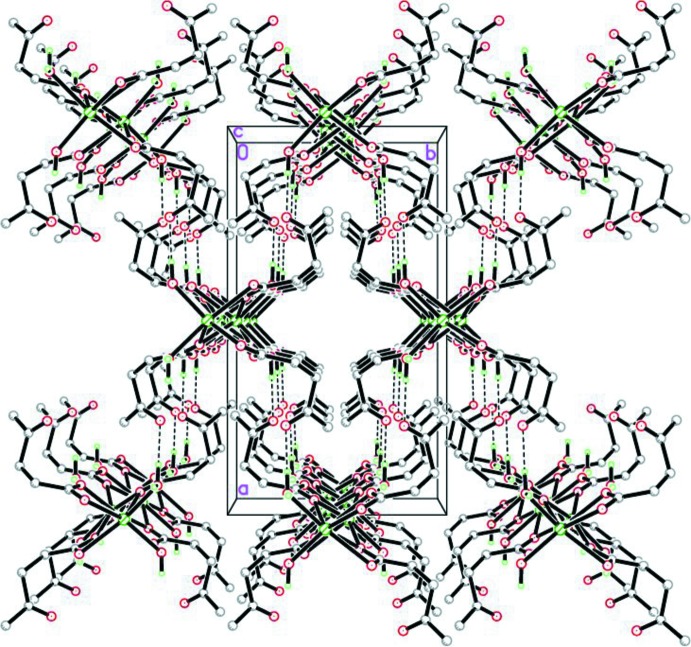
The three-dimensional hydrogen-bonded structure in the unit cell viewed along the *c* axis. Hydrogen-bonding inter­actions are shown as dashed lines.

In the crystal, the polymer chains are linked *via* inter-chain hydrogen bonds between the second H atom of the coordinating water mol­ecule and the carbonyl O atom of an adjacent chain (O4—H4*A*⋯O3^iv^), giving an overall three-dimensional structure (Fig. 3 ▸) [for symmetry code (iv), see Table 2 ▸]. To achieve this hydrogen-bonding inter­action, the levulinate mol­ecule is twisted [torsion angle C1—C2—C3—C4 = 73.2 (2)°].

## Database survey   

The Cu^2+^ levulinate structures represent examples of a very small number of metal levulinates in the crystallographic literature (Zubkowski *et al.*, 1997 ▸). Only one of these involves the levulinate ligand alone: a polymeric structure formed through carboxyl O-linked tetra­carboxyl­ate-bridged dimers, in which the copper atoms have nearly square-pyramidal coordination geometry. In the same report are the structures of three additional Cu^2+^ complexes with levulinate as well as other ligands: pyridine, 2,2′-bi­pyridine and tri­phenyl­phosphine. The crystal structures of two polymorphic forms of the analogous calcium acetate monohydrate salt are also known (Klop *et al.*, 1984 ▸; Van der Sluis *et al.*, 1987 ▸).

## Synthesis and crystallization   

Levulinic acid (1.160 g, 10.0 mmol) was added to a suspension of calcium hydroxide (0.370 g, 5.00 mmol) in 200 mL of deionized water in a beaker. The mixture was boiled with magnetic stirring on a hot plate to form a clear solution, then transferred to an evaporating dish and allowed to crystallize at room temperature. The product was collected under suction filtration, dried at 363 K for 24 h to give 1.455 g of calcium levulinate dihydrate as white needle-shaped crystals in 95% yield. Found: C, 39.02; H, 6.23; calculated for [Ca(C_5_H_7_O_3_)_2_(H_2_O)_2_]: C, 39.21; H, 5.92%. ^1^H NMR (DMSO-*d*6) δ 2.05 (3H, *s*), 2.19 (2H, *t*, *J* = 6.8 Hz), 2.54 (2H, *t*, *J* = 6.8 Hz). ^13^C NMR (DMSO-*d*6) δ 30.2, 31.5, 37.9, 179.6, 208.9. The single crystals for X-ray crystallographic analysis were grown by allowing a saturated solution of calcium levu­linate dihydrate in 20% methanol in water to stand at room temperature for five days.

## Refinement   

Crystal data, data collection and structure refinement details are summarized in Table 3 ▸. The C-bound H atoms were placed in calculated positions and allowed to ride on their carrier atoms: C—H = 0.93–0.97 Å with *U*_iso_(H) = 1.5*U*_eq_(C) for methyl H atoms and 1.2*U*_eq_(C) for other H atoms. The water H atoms were found using a Fourier map and were also allowed to ride in the refinement, O—H = 0.90 Å and with *U*_iso_(H) = 1.5*U*_eq_(O).

## Supplementary Material

Crystal structure: contains datablock(s) I. DOI: 10.1107/S2056989015006696/zs2328sup1.cif

Structure factors: contains datablock(s) I. DOI: 10.1107/S2056989015006696/zs2328Isup2.hkl

CCDC reference: 1057749

Additional supporting information:  crystallographic information; 3D view; checkCIF report

## Figures and Tables

**Table 1:** Selected bond lengths ()

Ca1O1^i^	2.3546(10)	Ca1O2^iii^	2.4820(10)
Ca1O1^ii^	2.3546(10)	Ca1O2	2.4820(11)
Ca1O4^iii^	2.4367(10)	Ca1O1	2.5989(10)
Ca1O4	2.4367(10)	Ca1O1^iii^	2.5990(10)

Symmetry codes: (i) 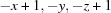; (ii) 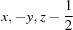; (iii) 
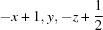
.

**Table 2:** Hydrogen-bond geometry (, )

*D*H*A*	*D*H	H*A*	*D* *A*	*D*H*A*
O4H4*A*O3^iv^	0.90	2.02	2.8568(15)	155
O4H4*B*O2^v^	0.90	1.87	2.7519(14)	168

Symmetry codes: (iv) 
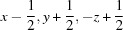
; (v) 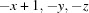.

**Table 3:** Experimental details

Crystal data	
Chemical formula	[Ca(C_5_H_7_O_3_)_2_(H_2_O)_2_]
*M*_r_	306.32
Crystal system, space group	Orthorhombic, *P* *b* *c* *n*
Temperature (K)	100
*a*, *b*, *c* ()	17.644(3), 9.9627(19), 7.8160(15)
*V* (^3^)	1373.9(5)
*Z*	4
Radiation type	Mo *K*
(mm^1^)	0.49
Crystal size (mm)	0.94 0.11 0.08

Data collection	
Diffractometer	Bruker SMART APEXII CCD area detector
Absorption correction	Multi-scan (*SADABS*; Bruker, 2005 ▸)
*T*_min_, *T*_max_	0.656, 0.963
No. of measured, independent and observed [*I* > 2(*I*)] reflections	11806, 1664, 1571
*R*_int_	0.020
(sin /)_max_ (^1^)	0.660

Refinement	
*R*[*F* ^2^ > 2(*F* ^2^)], *wR*(*F* ^2^), *S*	0.030, 0.083, 1.16
No. of reflections	1664
No. of parameters	88
H-atom treatment	H-atom parameters constrained
_max_, _min_ (e ^3^)	0.53, 0.54

Computer programs: *APEX2* and *SAINT* (Bruker, 2005 ▸), *SHELXS97*, *SHELXL97* and *SHELXTL* (Sheldrick, 2008 ▸) and *OLEX2* (Dolomanov *et al.*, 2009 ▸).

## supplementary crystallographic information

### Crystal data

**Table:**

[Ca(C_5_H_7_O_3_)_2_(H_2_O)_2_]	*F*(000) = 648
*M**_r_* = 306.32	*D*_x_ = 1.481 Mg m^−^^3^
Orthorhombic, *P**b**c**n*	Mo *K*α radiation, λ = 0.71073 Å
Hall symbol: -P 2n 2ab	Cell parameters from 8589 reflections
*a* = 17.644 (3) Å	θ = 2.3–30.5°
*b* = 9.9627 (19) Å	µ = 0.49 mm^−^^1^
*c* = 7.8160 (15) Å	*T* = 100 K
*V* = 1373.9 (5) Å^3^	Needle, colourless
*Z* = 4	0.94 × 0.11 × 0.08 mm

### Data collection

**Table:**

Bruker SMART APEXII CCD area-detector diffractometer	1664 independent reflections
Radiation source: fine-focus sealed tube	1571 reflections with *I* > 2σ(*I*)
Graphite monochromator	*R*_int_ = 0.020
φ and ω scans	θ_max_ = 28.0°, θ_min_ = 2.3°
Absorption correction: multi-scan (*SADABS*; Bruker, 2005)	*h* = −23→23
*T*_min_ = 0.656, *T*_max_ = 0.963	*k* = −13→13
11806 measured reflections	*l* = −10→10

### Refinement

**Table:**

Refinement on *F*^2^	Primary atom site location: structure-invariant direct methods
Least-squares matrix: full	Secondary atom site location: difference Fourier map
*R*[*F*^2^ > 2σ(*F*^2^)] = 0.030	Hydrogen site location: inferred from neighbouring sites
*wR*(*F*^2^) = 0.083	H-atom parameters constrained
*S* = 1.16	*w* = 1/[σ^2^(*F*_o_^2^) + (0.0384*P*)^2^ + 1.0525*P*] where *P* = (*F*_o_^2^ + 2*F*_c_^2^)/3
1664 reflections	(Δ/σ)_max_ < 0.001
88 parameters	Δρ_max_ = 0.53 e Å^−^^3^
0 restraints	Δρ_min_ = −0.54 e Å^−^^3^

### Special details

**Table:**

Geometry. All e.s.d.'s (except the e.s.d. in the dihedral angle between two l.s. planes) are estimated using the full covariance matrix. The cell e.s.d.'s are taken into account individually in the estimation of e.s.d.'s in distances, angles and torsion angles; correlations between e.s.d.'s in cell parameters are only used when they are defined by crystal symmetry. An approximate (isotropic) treatment of cell e.s.d.'s is used for estimating e.s.d.'s involving l.s. planes.
Refinement. Refinement of *F*^2^ against ALL reflections. The weighted *R*-factor *wR* and goodness of fit *S* are based on *F*^2^, conventional *R*-factors *R* are based on *F*, with *F* set to zero for negative *F*^2^. The threshold expression of *F*^2^ > σ(*F*^2^) is used only for calculating *R*-factors(gt) *etc*. and is not relevant to the choice of reflections for refinement. *R*-factors based on *F*^2^ are statistically about twice as large as those based on *F*, and *R*- factors based on ALL data will be even larger.

### Fractional atomic coordinates and isotropic or equivalent isotropic displacement parameters (Å^2^)

**Table:**

	*x*	*y*	*z*	*U*_iso_*/*U*_eq_	
Ca1	0.5000	0.04993 (4)	0.2500	0.00808 (12)	
O4	0.40994 (5)	0.20740 (10)	0.12520 (12)	0.0141 (2)	
H4A	0.3629	0.1929	0.1655	0.021*	
H4B	0.4086	0.1988	0.0107	0.021*	
O2	0.58523 (5)	−0.14668 (10)	0.21809 (12)	0.0123 (2)	
C4	0.71782 (8)	−0.37537 (14)	0.25360 (16)	0.0120 (3)	
O1	0.55856 (5)	−0.10067 (9)	0.48715 (12)	0.0115 (2)	
C1	0.58068 (7)	−0.18090 (13)	0.37294 (16)	0.0094 (2)	
C2	0.59849 (8)	−0.32410 (14)	0.42594 (18)	0.0149 (3)	
H2A	0.5507	−0.3682	0.4616	0.018*	
H2B	0.6324	−0.3215	0.5269	0.018*	
C3	0.63568 (8)	−0.40979 (13)	0.28835 (19)	0.0140 (3)	
H3A	0.6326	−0.5052	0.3232	0.017*	
H3B	0.6066	−0.3997	0.1807	0.017*	
O3	0.74945 (6)	−0.28329 (10)	0.32774 (13)	0.0166 (2)	
C5	0.75831 (8)	−0.46004 (14)	0.12409 (18)	0.0155 (3)	
H5A	0.8107	−0.4287	0.1123	0.023*	
H5B	0.7325	−0.4530	0.0135	0.023*	
H5C	0.7583	−0.5539	0.1618	0.023*	

### Atomic displacement parameters (Å^2^)

**Table:**

	*U*^11^	*U*^22^	*U*^33^	*U*^12^	*U*^13^	*U*^23^
Ca1	0.00891 (19)	0.00906 (19)	0.00626 (18)	0.000	0.00041 (11)	0.000
O4	0.0144 (5)	0.0187 (5)	0.0092 (4)	0.0031 (4)	0.0010 (3)	0.0003 (4)
O2	0.0133 (4)	0.0152 (5)	0.0084 (4)	0.0034 (4)	0.0011 (3)	0.0007 (4)
C4	0.0140 (6)	0.0107 (6)	0.0113 (6)	0.0030 (5)	−0.0017 (4)	0.0023 (5)
O1	0.0111 (4)	0.0140 (5)	0.0093 (4)	0.0021 (3)	0.0009 (3)	−0.0016 (4)
C1	0.0058 (5)	0.0125 (6)	0.0099 (6)	0.0001 (4)	−0.0001 (4)	0.0001 (5)
C2	0.0168 (6)	0.0136 (6)	0.0142 (6)	0.0041 (5)	0.0044 (5)	0.0032 (5)
C3	0.0136 (6)	0.0114 (6)	0.0172 (6)	0.0020 (5)	0.0007 (5)	−0.0009 (5)
O3	0.0163 (5)	0.0148 (5)	0.0187 (5)	0.0005 (4)	−0.0029 (4)	−0.0037 (4)
C5	0.0154 (6)	0.0153 (6)	0.0160 (6)	0.0011 (5)	0.0024 (5)	−0.0026 (5)

### Geometric parameters (Å, º)

**Table:**

Ca1—O1^i^	2.3546 (10)	C4—C3	1.5138 (19)
Ca1—O1^ii^	2.3546 (10)	O1—C1	1.2602 (16)
Ca1—O4^iii^	2.4367 (10)	O1—Ca1^i^	2.3546 (10)
Ca1—O4	2.4367 (10)	C1—C2	1.5185 (18)
Ca1—O2^iii^	2.4820 (10)	C2—C3	1.5218 (19)
Ca1—O2	2.4820 (11)	C2—H2A	0.9900
Ca1—O1	2.5989 (10)	C2—H2B	0.9900
Ca1—O1^iii^	2.5990 (10)	C3—H3A	0.9900
O4—H4A	0.8999	C3—H3B	0.9900
O4—H4B	0.8994	C5—H5A	0.9800
O2—C1	1.2599 (16)	C5—H5B	0.9800
C4—O3	1.2203 (17)	C5—H5C	0.9800
C4—C5	1.4988 (18)		
			
O1^i^—Ca1—O1^ii^	155.21 (5)	O2—Ca1—Ca1^iv^	73.02 (2)
O1^i^—Ca1—O4^iii^	78.38 (3)	O1—Ca1—Ca1^iv^	123.28 (3)
O1^ii^—Ca1—O4^iii^	85.69 (3)	O1^iii^—Ca1—Ca1^iv^	33.53 (2)
O1^i^—Ca1—O4	85.69 (3)	C1—Ca1—Ca1^iv^	97.28 (3)
O1^ii^—Ca1—O4	78.39 (3)	C1^iii^—Ca1—Ca1^iv^	58.53 (3)
O4^iii^—Ca1—O4	99.84 (5)	O1^i^—Ca1—Ca1^i^	37.57 (2)
O1^i^—Ca1—O2^iii^	79.39 (3)	O1^ii^—Ca1—Ca1^i^	153.97 (2)
O1^ii^—Ca1—O2^iii^	121.53 (3)	O4^iii^—Ca1—Ca1^i^	76.76 (2)
O4^iii^—Ca1—O2^iii^	149.65 (3)	O4—Ca1—Ca1^i^	123.15 (2)
O4—Ca1—O2^iii^	98.81 (4)	O2^iii^—Ca1—Ca1^i^	73.02 (2)
O1^i^—Ca1—O2	121.53 (3)	O2—Ca1—Ca1^i^	84.41 (2)
O1^ii^—Ca1—O2	79.39 (3)	O1—Ca1—Ca1^i^	33.53 (2)
O4^iii^—Ca1—O2	98.81 (4)	O1^iii^—Ca1—Ca1^i^	123.28 (3)
O4—Ca1—O2	149.65 (3)	C1—Ca1—Ca1^i^	58.53 (3)
O2^iii^—Ca1—O2	75.78 (5)	C1^iii^—Ca1—Ca1^i^	97.28 (3)
O1^i^—Ca1—O1	71.10 (4)	Ca1—O4—H4A	111.0
O1^ii^—Ca1—O1	124.87 (4)	Ca1—O4—H4B	110.8
O4^iii^—Ca1—O1	80.03 (3)	H4A—O4—H4B	108.0
O4—Ca1—O1	156.41 (3)	C1—O2—Ca1	94.50 (8)
O2^iii^—Ca1—O1	73.36 (3)	O3—C4—C5	121.72 (13)
O2—Ca1—O1	51.33 (3)	O3—C4—C3	121.53 (12)
O1^i^—Ca1—O1^iii^	124.87 (4)	C5—C4—C3	116.75 (12)
O1^ii^—Ca1—O1^iii^	71.10 (4)	C1—O1—Ca1^i^	152.89 (9)
O4^iii^—Ca1—O1^iii^	156.41 (3)	C1—O1—Ca1	89.09 (8)
O4—Ca1—O1^iii^	80.03 (3)	Ca1^i^—O1—Ca1	108.90 (4)
O2^iii^—Ca1—O1^iii^	51.33 (3)	O2—C1—O1	121.91 (12)
O2—Ca1—O1^iii^	73.36 (3)	O2—C1—C2	120.21 (12)
O1—Ca1—O1^iii^	109.48 (5)	O1—C1—C2	117.82 (11)
O1^i^—Ca1—C1	95.60 (4)	O2—C1—Ca1	59.55 (7)
O1^ii^—Ca1—C1	104.27 (4)	O1—C1—Ca1	64.87 (7)
O4^iii^—Ca1—C1	93.35 (4)	C2—C1—Ca1	161.22 (9)
O4—Ca1—C1	166.72 (4)	C1—C2—C3	115.05 (11)
O2^iii^—Ca1—C1	68.56 (4)	C1—C2—H2A	108.5
O2—Ca1—C1	25.95 (3)	C3—C2—H2A	108.5
O1—Ca1—C1	26.04 (3)	C1—C2—H2B	108.5
O1^iii^—Ca1—C1	88.47 (4)	C3—C2—H2B	108.5
O1^i^—Ca1—C1^iii^	104.27 (4)	H2A—C2—H2B	107.5
O1^ii^—Ca1—C1^iii^	95.60 (4)	C4—C3—C2	114.37 (11)
O4^iii^—Ca1—C1^iii^	166.72 (4)	C4—C3—H3A	108.7
O4—Ca1—C1^iii^	93.35 (4)	C2—C3—H3A	108.7
O2^iii^—Ca1—C1^iii^	25.95 (3)	C4—C3—H3B	108.7
O2—Ca1—C1^iii^	68.56 (4)	C2—C3—H3B	108.7
O1—Ca1—C1^iii^	88.47 (4)	H3A—C3—H3B	107.6
O1^iii^—Ca1—C1^iii^	26.04 (3)	C4—C5—H5A	109.5
C1—Ca1—C1^iii^	73.51 (5)	C4—C5—H5B	109.5
O1^i^—Ca1—Ca1^iv^	153.97 (2)	H5A—C5—H5B	109.5
O1^ii^—Ca1—Ca1^iv^	37.57 (2)	C4—C5—H5C	109.5
O4^iii^—Ca1—Ca1^iv^	123.15 (2)	H5A—C5—H5C	109.5
O4—Ca1—Ca1^iv^	76.76 (2)	H5B—C5—H5C	109.5
O2^iii^—Ca1—Ca1^iv^	84.41 (2)		
			
O1^i^—Ca1—O2—C1	−2.30 (9)	O1^i^—Ca1—C1—O2	178.03 (8)
O1^ii^—Ca1—O2—C1	163.32 (8)	O1^ii^—Ca1—C1—O2	−16.92 (8)
O4^iii^—Ca1—O2—C1	79.43 (8)	O4^iii^—Ca1—C1—O2	−103.32 (8)
O4—Ca1—O2—C1	−153.18 (8)	O4—Ca1—C1—O2	83.05 (17)
O2^iii^—Ca1—O2—C1	−70.07 (7)	O2^iii^—Ca1—C1—O2	101.76 (8)
O1—Ca1—O2—C1	9.82 (7)	O1—Ca1—C1—O2	−162.35 (12)
O1^iii^—Ca1—O2—C1	−123.41 (8)	O1^iii^—Ca1—C1—O2	53.14 (8)
C1^iii^—Ca1—O2—C1	−96.30 (8)	C1^iii^—Ca1—C1—O2	74.78 (8)
Ca1^iv^—Ca1—O2—C1	−158.49 (8)	Ca1^iv^—Ca1—C1—O2	20.71 (8)
Ca1^i^—Ca1—O2—C1	3.79 (7)	Ca1^i^—Ca1—C1—O2	−175.57 (9)
O1^i^—Ca1—O1—C1	159.32 (9)	O1^i^—Ca1—C1—O1	−19.62 (9)
O1^ii^—Ca1—O1—C1	−42.09 (8)	O1^ii^—Ca1—C1—O1	145.43 (7)
O4^iii^—Ca1—O1—C1	−119.65 (7)	O4^iii^—Ca1—C1—O1	59.02 (7)
O4—Ca1—O1—C1	148.55 (9)	O4—Ca1—C1—O1	−114.60 (15)
O2^iii^—Ca1—O1—C1	75.10 (7)	O2^iii^—Ca1—C1—O1	−95.90 (7)
O2—Ca1—O1—C1	−9.79 (7)	O2—Ca1—C1—O1	162.35 (12)
O1^iii^—Ca1—O1—C1	37.99 (6)	O1^iii^—Ca1—C1—O1	−144.52 (6)
C1^iii^—Ca1—O1—C1	53.67 (9)	C1^iii^—Ca1—C1—O1	−122.87 (9)
Ca1^iv^—Ca1—O1—C1	3.63 (8)	Ca1^iv^—Ca1—C1—O1	−176.95 (7)
Ca1^i^—Ca1—O1—C1	159.32 (9)	Ca1^i^—Ca1—C1—O1	−13.22 (6)
O1^i^—Ca1—O1—Ca1^i^	0.0	O1^i^—Ca1—C1—C2	83.2 (3)
O1^ii^—Ca1—O1—Ca1^i^	158.59 (4)	O1^ii^—Ca1—C1—C2	−111.8 (3)
O4^iii^—Ca1—O1—Ca1^i^	81.03 (4)	O4^iii^—Ca1—C1—C2	161.8 (3)
O4—Ca1—O1—Ca1^i^	−10.77 (10)	O4—Ca1—C1—C2	−11.8 (4)
O2^iii^—Ca1—O1—Ca1^i^	−84.22 (4)	O2^iii^—Ca1—C1—C2	6.9 (3)
O2—Ca1—O1—Ca1^i^	−169.10 (6)	O2—Ca1—C1—C2	−94.8 (3)
O1^iii^—Ca1—O1—Ca1^i^	−121.33 (4)	O1—Ca1—C1—C2	102.8 (3)
C1—Ca1—O1—Ca1^i^	−159.32 (9)	O1^iii^—Ca1—C1—C2	−41.7 (3)
C1^iii^—Ca1—O1—Ca1^i^	−105.65 (4)	C1^iii^—Ca1—C1—C2	−20.0 (3)
Ca1^iv^—Ca1—O1—Ca1^i^	−155.69 (2)	Ca1^iv^—Ca1—C1—C2	−74.1 (3)
Ca1—O2—C1—O1	−18.87 (13)	Ca1^i^—Ca1—C1—C2	89.6 (3)
Ca1—O2—C1—C2	158.21 (10)	O2—C1—C2—C3	11.36 (18)
Ca1^i^—O1—C1—O2	150.77 (13)	O1—C1—C2—C3	−171.45 (11)
Ca1—O1—C1—O2	17.93 (12)	Ca1—C1—C2—C3	95.1 (3)
Ca1^i^—O1—C1—C2	−26.4 (2)	O3—C4—C3—C2	−2.20 (18)
Ca1—O1—C1—C2	−159.21 (10)	C5—C4—C3—C2	177.69 (12)
Ca1^i^—O1—C1—Ca1	132.83 (18)	C1—C2—C3—C4	73.21 (15)

Symmetry codes: (i) −*x*+1, −*y*, −*z*+1; (ii) *x*, −*y*, *z*−1/2; (iii) −*x*+1, *y*, −*z*+1/2; (iv) −*x*+1, −*y*, −*z*.

### Hydrogen-bond geometry (Å, º)

**Table:**

*D*—H···*A*	*D*—H	H···*A*	*D*···*A*	*D*—H···*A*
O4—H4*A*···O3^v^	0.90	2.02	2.8568 (15)	155
O4—H4*B*···O2^iv^	0.90	1.87	2.7519 (14)	168

Symmetry codes: (iv) −*x*+1, −*y*, −*z*; (v) *x*−1/2, *y*+1/2, −*z*+1/2.
